# Chemical composition of food induces plasticity in digestive morphology in larvae of *Rana temporaria*

**DOI:** 10.1242/bio.048041

**Published:** 2019-12-18

**Authors:** Katharina Ruthsatz, Lisa Marie Giertz, Dominik Schröder, Julian Glos

**Affiliations:** Department of Biology, Institute for Zoology, University of Hamburg, Martin-Luther-King Platz 3, 20146 Hamburg, Germany

**Keywords:** Adaptability, Climate change, Gut length, Oral papillae, Protein content, Nutrient content

## Abstract

Food conditions are changing due to anthropogenic activities and natural sources and thus, many species are exposed to new challenges. Animals might cope with altered quantitative and qualitative composition [i.e. variable protein, nitrogen (N) and energy content] of food by exhibiting trophic and digestive plasticity. We examined experimentally whether tadpoles of the common frog (*Rana temporaria*) exhibit phenotypic plasticity of the oral apparatus and intestinal morphology when raised on a diet of either low (i.e. *Spirulina* algae) or high protein, N and energy content (i.e. *Daphnia pulex*). Whereas intestinal morphology was highly plastic, oral morphology did not respond plastically to different chemical compositions of food. Tadpoles that were fed food with low protein and N content and low-energy density developed significantly longer guts and a larger larval stomachs than tadpoles raised on high protein, N and an energetically dense diet, and developed a different intestinal surface morphology. Body sizes of the treatment groups were similar, indicating that tadpoles fully compensated for low protein, N and energy diet by developing longer intestines. The ability of a species, *R. temporaria*, to respond plastically to environmental variation indicates that this species might have the potential to cope with new conditions during climate change.

## Abstract (precis)

**Summary:** Tadpoles of the common frog (*Rana temporaria*) exhibit phenotypic plasticity of the intestinal morphology but not of the oral apparatus when raised on diets of different chemical compositions.

## INTRODUCTION

Many species are experiencing sustained environmental change mainly due to anthropogenic activities (e.g. climate change, pollution and habitat fragmentation), but also from natural sources (Chevin et al., 2010; Noyes et al., 2009; Dantzer et al., 2014). Animals can cope with changing environmental conditions by either migration, genetic adaptation or in the evolution of phenotypic plasticity (Agrawal, 2001; Hoffmann and Sgró 2011; Seebacher et al., 2015). Phenotypic plasticity is the ability of a single genotype to produce more than one phenotype, e.g. a form of morphology, behavior, development and physiological state in response to environmental conditions (West-Eberhard, 1989; Newman, 1992; Agrawal, 2001; reviewed in Miner et al., 2005), and is adaptive in heterogeneous environments (Ghalambor et al., 2007; Naya et al., 2007). However, phenotypic plasticity for some traits may not have an adaptive value or even be maladaptive (reviewed in Agrawal, 2001; Pigliucci, 2005, reviewed in Zaldúa and Naya, 2014). Furthermore, there can also be fitness costs (and limits) of being phenotypically plastic above and beyond the costs of producing a particular phenotype (DeWitt et al., 1998). Therefore, the benefits of a plastic response to an environmental change must outweigh the costs to be adaptive (van Tienderen, 1991; Relyea, 2001a; Van Buskirk, 2001).

One major challenge of climate change for animals is altered food conditions as a result of either direct effects on food availability or quality, or through a cascade of indirect effects such as ambient temperature changes, predator introduction or pollution resulting in different growth rates of different food types (reviewed in Carreira et al., 2016; reviewed in Rowland et al., 2016; Norlin et al., 2016; Eby et al., 2006). Accordingly, altered food conditions (i.e. food quantity and chemical composition) due to climate change may lead to new constraints for both food intake and digestion, which animals might cope with through exhibiting trophic and digestive plasticity (Ke et al., 2008; Stoler and Relyea, 2013; Carreira et al., 2016). Digestive plasticity is found in many animal taxa in response to varying food quality and quantity (McWilliams and Karasov, 2001; Stevens and Hume, 2004; Cramp and Franklin, 2003; Secor, 2005), particularly regarding phenotypic characters associated with food intake (i.e. oral morphology) food digestion (i.e. digestive system, enzyme activities) (e.g. Starck, 1996; McWilliams and Karasov, 2001; Relyea and Auld, 2004) and nutrient transport (Sabat et al., 1995; Secor and Diamond, 2000; Castañeda et al., 2006).

Across vertebrate taxa, and consistent among species of mammals, birds, reptiles, fish and amphibians, herbivores exhibit longer digestive tracts than carnivores due to differences in food quality (reviewed in Stevens and Hume, 2004; reviewed in Naya and Bozinovic, 2004; reviewed in Karasov and Hume, 2011). These differences in relative gut length can be explained by the ‘optimal digestion theory’ (Sibly, 1981; Relyea and Auld, 2004; Venesky et al., 2013). It states that animals that consume food of lower protein and energy content and more non-digestible material, as is the case for plant-based food (Raubenheimer et al., 2009), have longer digestive systems, since these lead to longer gut passage times of food and therefore to improved digestive efficiency (Sibly, 1981; Yang and Joern, 1994; Relyea and Auld, 2004). Furthermore, longer guts are needed to avoid a decrease in assimilation, since animals ingest more food that is low in protein, nitrogen (N) and energy content in order to maintain the amount of food assimilated with a similar digestive efficiency (Carreira et al., 2016).

In amphibians, much work has investigated the effects of predation, competition and diet quantity on larval and adult digestive morphology and physiology, indicating a decrease in intestinal performance, size, mass and surface during periods of fasting or hypophagy (e.g. Karasov and Diamond, 1983; Relyea and Auld, 2004; Cramp and Franklin, 2005; Cramp and Franklin, 2005; Castañeda et al., 2006; Seliverstova and Prutskova, 2012). Lower food quantity induced longer and heavier intestines and wider mouths in tadpoles of the wood frog (*Lithobates sylvaticus*, Relyea, 2002; Relyea and Auld, 2004, 2005) and increased the surface of the larval stomach (i.e. *Manicotto glandulare*) in the red-eyed tree frog (*Agalychnis callidryas*, Bouchard et al., 2016). The very early studies of Babak (1905) and Yung (1904) demonstrated that tadpoles show digestive plasticity in response to different food and particularly protein sources. Further, experimental studies showed that a tree frog (*Rhacophorus arboreus)* and a spadefoot toad (*Scaphiopus multiplicatus*) raised with purely herbivorous diets developed longer intestines than tadpoles on a purely carnivorous diet (Horiuchi and Koshida, 1989; Pfennig, 1992a,b; Relyea and Auld, 2004). Further, intestine length and mouth size of *L. sylvaticus* larvae increased and decreased respectively with decreasing N content (Stoler and Relyea, 2013). Studies on the influence of chemical composition of food on other parts of the digestive system that are relevant for food storage before the gut passage (i.e. the larval stomach, *Manicotto glandulare*; McDiarmid and Altig, 1999; Haas et al., 2014) or on the efficiency of food uptake via the intestinal surfaces (i.e. microvilli; Shi and Ishizuya-Oka, 1996; McDiarmid and Altig, 1999) are so far lacking.

Since larval amphibians will experience variability in resource quantity and composition due to climate change (e.g. increased competition due to pond desiccation, Mogali et al., 2016; higher energy demand due to temperature increase, Carreira et al., 2016; impacts of water quality on food items, Schmeller, 2018; Smalling et al., 2019), this study aims to investigate whether tadpoles of the common frog (*Rana temporaria*) show plasticity in their digestive morphology (i.e. oral and intestinal structures) in response to different food chemistry (i.e. high and low N content and energy density). Larvae of *R. temporaria* are known to react highly plastically to variation in environmental factors (e.g. Merilä et al., 2000, 2004) and are considered to be more plastic during larval stage than other species (Laurila and Kujasalo, 1999). Several studies demonstrated that the capacity for digestive plasticity allows for higher growth efficiency and to compensate for shorter growth periods in high-latitude *R. temporaria* tadpoles (Lindgren and Laurila, 2005; Orizaola et al., 2014). However, studies investigating the capacity for digestive plasticity in response to different food quantity or different chemical compositions of food are so far lacking. We examined experimentally whether tadpoles of the common frog (*R. temporaria*) exhibit phenotypic plasticity of the oral apparatus and intestinal morphology when raised on a diet of either low (i.e. *Spirulina* algae, cyanobacteria) or high protein, N and energy content (i.e. *Daphnia pulex,* Crustacea, Cladoraecea) and therefore, might be able to react to changes in food composition associated with climate change. The following hypotheses were tested: (1) since animals consuming low protein, N and energy diet need to ingest a larger amount of food to meet their energetic requirements, we predicted larger stomachs and longer intestines in animals consuming a low N and energy diet (i.e. *Spirulina*) than in animals consuming a high N and energetically dense diet (i.e. *Daphnia*). (2) As density and length of intestinal microstructures (i.e. microvilli) on the intestinal surface are positively correlated to protein content of the diet, we expected a significant difference in intestinal microstructures related to nutrient uptake in low and high N diet. (3) Given that the oral apparatus of tadpoles is used for mechanical breakdown of the food, we predicted no significant change in oral structures when animals were fed with diets contrasting in its chemical composition but with a similar mechanical properties.

## RESULTS

### Body size

**Fig. 1.**
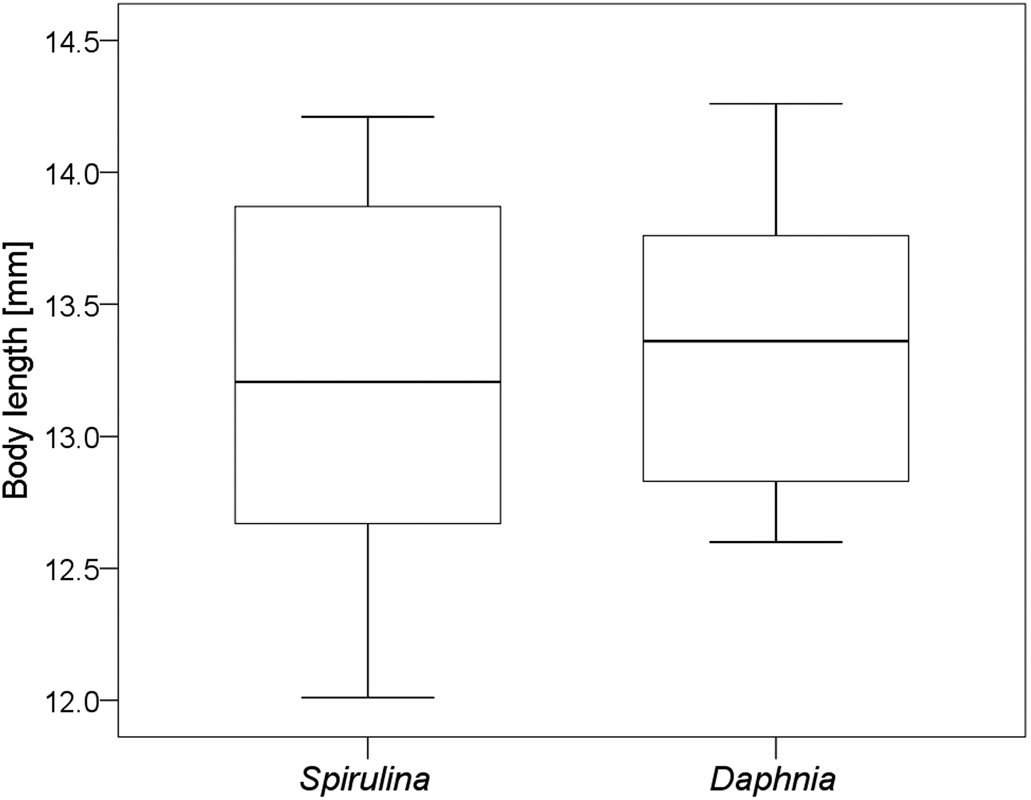
**Body sizes of *R. temporaria* tadpoles under low protein, N and energy content (*Spirulina*) and high protein, N and energy content food conditions (*Daphnia*).** Shown are medians, 25% and 75% percentiles (boxes) and minimum and maximum values (whiskers). No statistical difference between the two treatments.

Tadpoles that were fed *Spirulina* (low N and energy food) and *Daphnia* (high N and energy food) had similar growth and developmental rates. Body length at Gosner stage 36 of *Spirulina*-fed tadpoles (13.2±0.8 mm, mean±s.d.) did not significantly differ to body length in *Daphnia*-fed tadpoles (13.3±0.6 mm) (Fig. 1; Mann–Whitney *U*-test: Z=−1.55, *P*=0.33, *n*=16).

### Intestinal structures

**Fig. 2.**
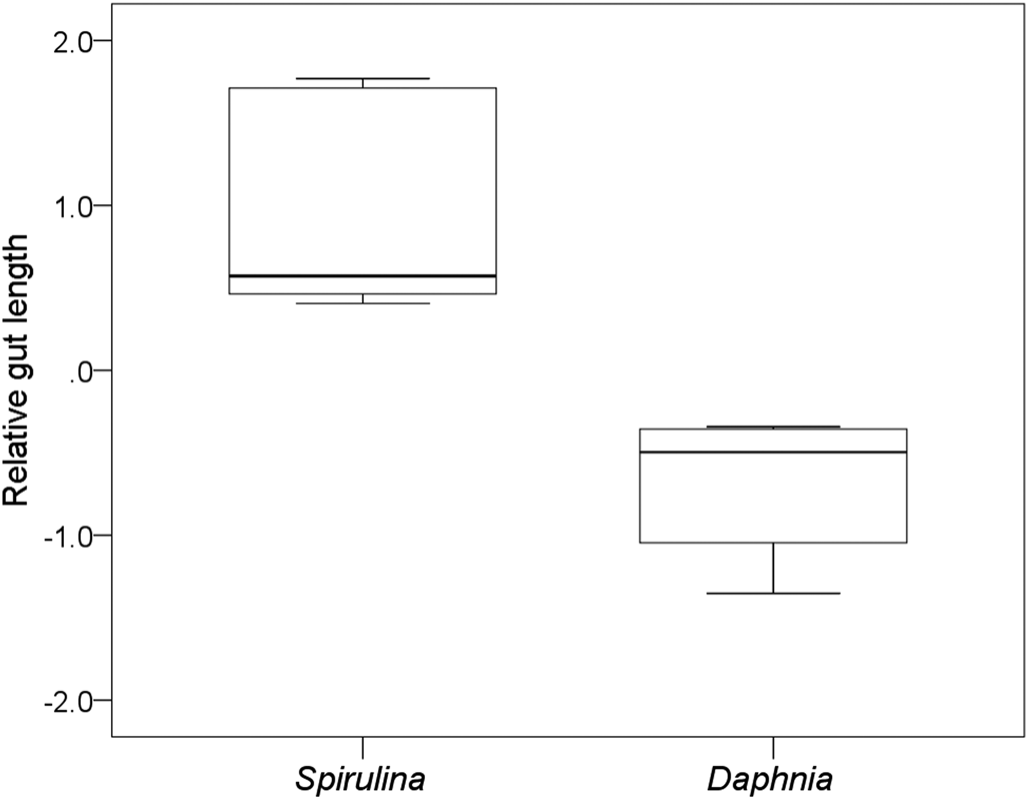
**Relative gut length.** Residuals of gut length versus body length of *R. temporaria* tadpoles under low protein, N and energy content (*Spirulina*) and high protein, N and energy content food conditions (*Daphnia*). Shown are medians, 25% and 75% percentiles (boxes) and minimum and maximum values (whiskers). The guts of *Spirulina*-fed tadpoles were significantly longer than those of *Daphnia*-fed tadpoles (*U*-test: *n*=14, *P*<0.001).

**Fig. 3.**
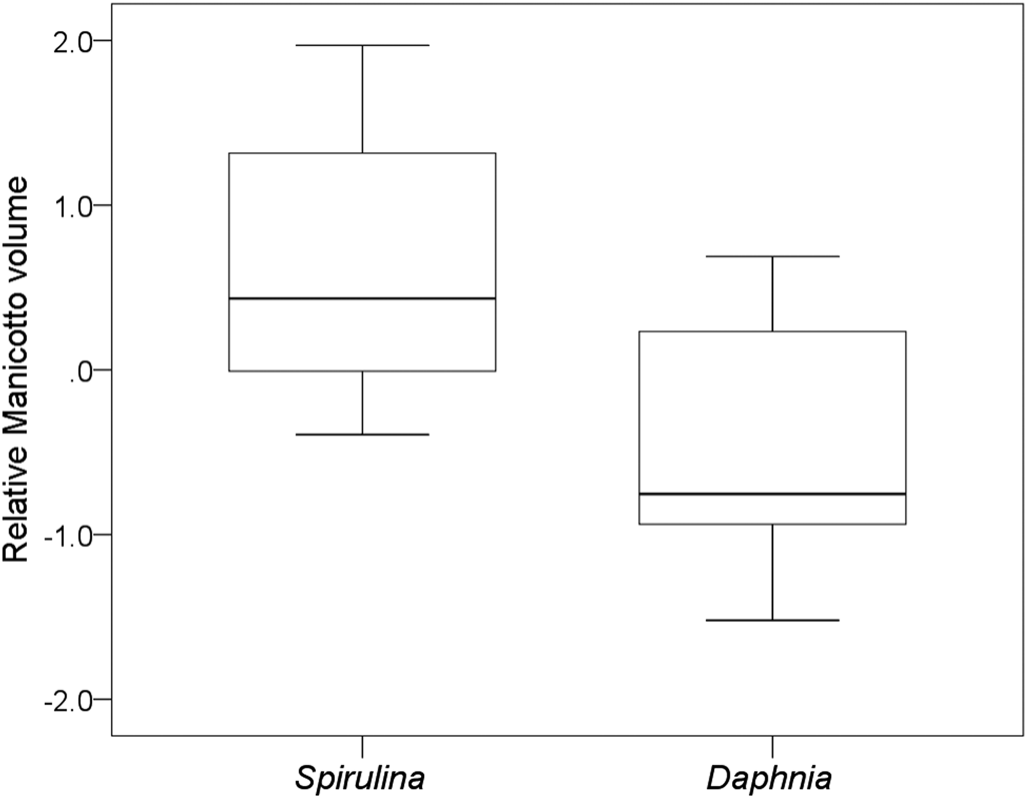
**Relative volume of the larval stomach *Manicotto glandulare*.** Residuals of *Manicotto glandulare* volume versus body length of *R. temporaria* tadpoles under low protein, N and energy content (*Spirulina*) and high protein, N and energy content food conditions (*Daphnia*). Shown are medians, 25% and 75% percentiles (boxes) and minimum and maximum values (whiskers). The *Manicotto* of *Spirulina*-fed tadpoles were significantly larger than of *Daphnia*-fed tadpoles (*U*-test: *n*=14, *P*<0.04).

There were considerable differences in the size of the intestinal tract between the treatments. The gut of tadpoles of the *Spirulina* treatment was 167% of the length of tadpoles that were fed *Daphnia* (absolute gut length: 171.4±33.2 mm versus 101.9±22.8 mm; mean±s.d.) (Mann–Whitney *U*-test on relative gut length: Z=−3.1, *N*=14, *P*=0.001; Fig. 2). Also, the volume of the larval stomach, *Manicotto glandulare*, in tadpoles of the *Spirulina* treatment was about twice as large as in tadpoles of the *Daphnia* treatment (absolute volume of *Manicotto glandulare*: 5.4±2.4 mm^2^ versus 2.8±2.3 mm^2^) (Mann–Whitney *U*-test on relative absolute volume of *Manicotto glandulare*: Z=−2.1, *N*=14, *P*=0.04; Fig. 3). However, there was no statistical difference in the diameter of the gut between both treatments (absolute gut diameter: 1.1±0.3 mm versus 1.0±0.2 mm) (Mann–Whitney *U*-test on relative gut diameter: Z=0.0, *N*=14, *P*=1.00).

### Intestinal microstructures

**Fig. 4.**
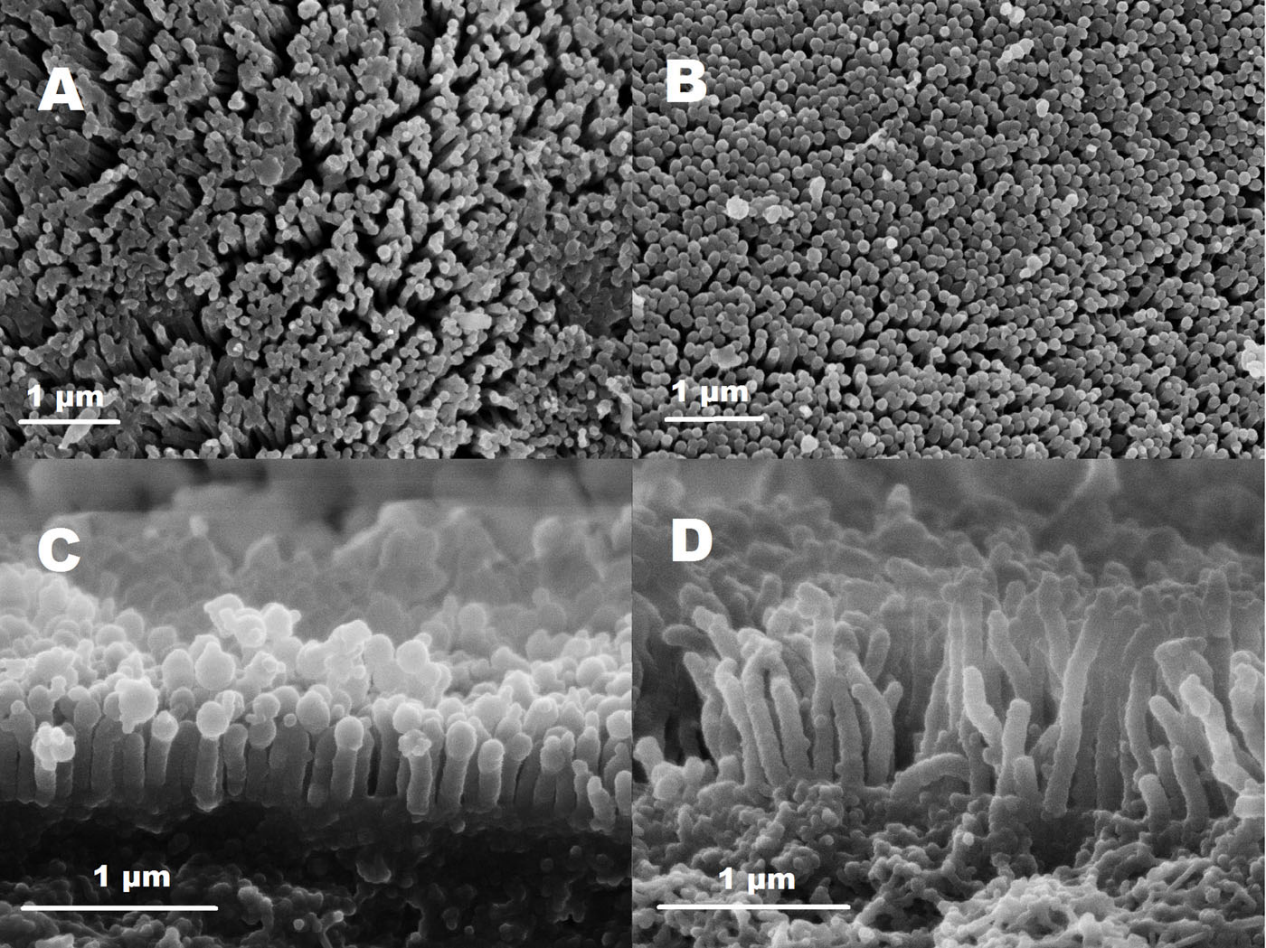
**Intestinal microstrcutures (microvilli seam) of larval *R. temporaria*.** Intestinal microstructures (microvilli seam) of *R. temporaria* tadpoles under high protein, N and energy content (*Daphnia;* A,C) and low protein, N and energy content food conditions (*Spirulina*; B,D). Shown are pictures taken by electron microscopy at 2600× (A,B) and 47,000× magnification (C,D).

The structures of the intestinal surface appeared to be considerably different between the two individuals measured for each food treatment (Fig. 4), although this could not be confirmed statistically due to only two replicates per treatment (Table 1). The density of microvilli was higher in the *Spirulina* group (49.2 and 50.0 microvilli/µm^2^) than in the *Daphnia* group (38.7 and 39.5). Individual microvilli were half as long in the *Daphnia* treatment (0.5 and 0.7 µm) compared to the *Spirulina* treatment (1.2 and 1.4 µm).

**Table 1.**
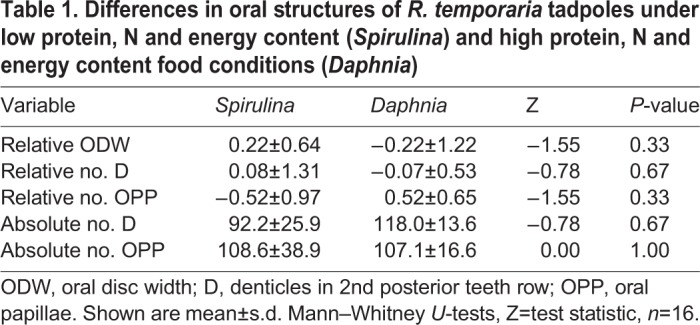
**Differences in oral structures of *R. temporaria* tadpoles under low protein, N and energy content (*Spirulina*) and high protein, N and energy content food conditions (*Daphnia*)**

### Oral structures

There were no significant statistical differences between the treatment groups in any of the oral structures, independent of whether the variables were adjusted to body size differences or not (Table 1).

## DISCUSSION

Gut plasticity is widespread in anuran larvae, with changes in intestinal length associated with competition (Bouchard et al., 2016), food quantity (Carabio et al., 2017), cold temperature (Lindgren and Laurila, 2005; Castañeda et al., 2006), the threat of predation (Relyea and Auld, 2004) and food quality (Stoler and Relyea, 2013). In this study, we demonstrate that differences in the chemical composition of food can induce dramatic effects on the intestinal system of tadpoles. Furthermore, this is the first study investigating diet-induced plasticity of the intestinal system in its entirety (i.e. oral structures, larval stomach, gut and intestinal surface microstructures). Tadpoles of *R. temporaria* fed with food of lower N content and lower energy density had longer guts, larger larval stomachs and more and larger microvilli on the intestinal surface than tadpoles fed with food of higher N content and energy density (hypotheses 1 and 2 were confirmed). However, there was no such plasticity in their oral apparatus, i.e. in structures related to food ingestion (hypothesis 3 was confirmed).

### High phenotypic plasticity in intestinal morphology

In recent years, an increasing number of studies on digestive plasticity in small ectotherm vertebrates such as amphibians have been published demonstrating that these organisms are able to adjust their digestive traits in response to changes in external conditions (reviewed in Naya et al., 2007). The phenotypic plasticity of intestinal structures in *R. temporaria* related to the chemical composition of food was very pronounced and affected a variety of intestinal structures, namely the *Manicotto glandulare* volume, intestinal length and microvilli morphology. Although the number of specimens in our study is limited and, thus, the data for intestinal microstructures could only be analyzed descriptively, the present results suggest a general effect of food chemical composition on the intestinal microstructures (i.e. microvilli length and density) and is in accordance with literature on other ectothermic taxa and adult amphibians (e.g. Emelyanova et al., 2004; Seliverstova and Prutskova, 2012; Cramp and Franklin, 2003; Cramp and Franklin, 2005).

Digestive plasticity is suggested to correlate with digestion efficiency (Sibly, 1981) and thus is likely to affect different processes important in nutrient uptake, such as the storage of food before assimilation (McDiarmid and Altig, 1999; Haas et al., 2014), and an increase in nutrient assimilation via increased gut passage time and intestinal surface area. The latter is particularly important, as digestion in tadpoles is supported by microbial fermentation of intestinal bacteria (Altig and Johnston, 1989). A large part of the energy in particular in low N, plant-based (or cyanobacteria-based) diets is in the form of cellulose (or murein in *Spirulina*) that no vertebrate can digest without its microbiome (Schmidt-Nielsen, 1975; Das, 1995; Karasov and Hume, 2011; McWilliams and Karasov, 2001). As microbial fermentation is very slow (Zimmerman and Tracey, 1989; Das, 1995) and needs a large volume, longer retention times in longer digestive tracts in *Spirulina* algae tadpoles supposedly lead to more effective utilization of energy in low-protein high-carbohydrate food. This phenotypic plasticity was triggered by differences in the chemical composition of the food rather than its mechanical texture as both *Spirulina* and *Daphnia* were fed in powdered form.

### No phenotypic plasticity in oral structures

Oral structures, as studied here, are related to food ingestion (i.e. oral disc width, denticles) and chemosensory aspects in feeding (i.e. oral papillae) (Thibaudeau and Altig, 1988; McDiarmid and Altig, 1999; Vences et al., 2002). Oral structures are known to be highly plastic characteristics within other tadpole species when the texture and size of food are different, causing different mechanical demands on oral structures, such as the size of the oral disc or properties of the tadpoles' teeth and beak (Pfennig and Murphy, 2002). However, when food sources were experimentally adjusted in this study to have equal mechanical textures (were powdered), but differed only in chemical composition, no phenotypic plasticity was found in oral structures in *R. temporaria* tadpoles. This indicates that the chemical composition of the food, i.e. protein and energy content, is not important in inducing phenotypic plasticity. Nonetheless, Pfennig and Murphy (2002) demonstrated that tadpoles of two closely related spadefoot toads (*Spea bombifrons* and *Spea multiplicata*) respond plastically to differences in diet (i.e. omnivore versus carnivore) in their oral morphologies (i.e. keratinized mouthparts, denticle rows and jaw muscles). Consequently, we consider that also tadpoles of *R. temporaria* might respond to different diets by exhibiting plasticity in oral morphology when both chemical composition and mechanical texture of the diet differs.

### Digestive plasticity and fitness consequences

The digestive tract represents a functional link between energy intake and energy allocation, and thus, gut plasticity is considered a trait with great influence on larval and post-metamorphic growth (Bouchard et al., 2016) and thus has important implications on animal performance and fitness (Naya et al., 2007). In this study, both phenotypes with different-sized intestines reached a late larval stage (i.e. Gosner stage 36, Gosner, 1960) at the same time and at the same body size. These variables, size of metamorphs and larval duration, are linked to individual fitness (Berven, 1990). Animals that reach metamorphosis earlier but at the same size, and animals with equally long larval development but that are larger at metamorphosis, are known to have a higher survival probability to the next season (Berven and Gill, 1983; Smith, 1987; Berven, 1990), to reproduce earlier (Smith, 1987; Berven, 1990; Scott, 1994), and are larger at the time of reproduction (Berven and Gill, 1983; Smith, 1987; Berven, 1990; Semlitsch et al., 1988; Scott, 1994). This study indicates that there may be no fitness differences between phenotypes that develop under different food conditions and hence that the observed phenotypic plasticity is adaptive. Therefore, tadpoles of *R. temporaria* might completely balance low N and energy food, despite the supposedly higher energetic costs of generation and maintenance of larger intestines. However, there are no studies so far demonstrating that plasticity in general and in digestive morphology actually increases fitness. Consequently, further studies are required to investigate how a plastic response in digestive morphology affects fitness in later life stages. Nevertheless, animals could have the same size, but differ in overall body composition (e.g. percent water in tissues) and body condition (i.e. size of energy stores).

The differences in gut length could also persist past metamorphosis and impact feeding behavior and physiology in juveniles, although anuran guts are completely remodeled during metamorphosis (Shi, 2000; Tata, 2006). Such carry-over effects of digestive plasticity have lately been demonstrated for froglets that have been exposed to different food quantities during larval stage (Bouchard et al., 2016).

### Ecological relevance of phenotypic plasticity in *R. temporaria*

*Rana temporaria* is seen as a model system for a very ecologically variable and flexible species (Laurila and Kujasalo, 1999). Its distribution area extends from the Iberian peninsula to western Siberia (Sillero et al., 2014); it occurs in altitudes from sea level to >2,500 m above sea level (Grossenbacher et al., 1988), and it reproduces in different water types, from small puddles to lakes (Schlüpmann and Günther, 1996). Accordingly, very variable abiotic (e.g. temperature, hydroperiod) and biotic factors (e.g. food quality and chemical composition) influence larval growth and development in *R. temporaria* populations. Among the explanations for this high ecological variability are a generally wide ecological niche, a high potential of adaptations of populations to local conditions (Drakulić et al., 2016) and a high potential for phenotypic plasticity in morphology (e.g. as a reaction to predation, Relyea, 2001a,b), and plasticity in important life history variables such as developmental and growth rate (e.g. depending on hydroperiod, Laurila et al., 2002; Merilä et al., 2000, 2004; depending on ambient temperature, Ruthsatz et al., 2018a,b). Our study indicates that this plasticity of intestinal morphology is another type of phenotypic plasticity that contributes to the high ecological flexibility of species. Tadpoles of *R. temporaria* adjust their intestinal morphology according to the available food source to maximize the efficiency of energy input, and accordingly are able to grow and develop successfully under a range of different food conditions in a wide variety of habitats. In addition to the capacity for digestive plasticity, plasticity in trophic morphology could reduce intraspecific and interspecific competition for food if it allows for a variation in the use of food sources in *R. temporaria* (Smith, 1987; Pfennig, 1992b; Walls et al., 1993; Miner et al., 2005; Ke et al., 2008).

## Conclusions

Climate change exposes wildlife to an array of environmental changes that arise from anthropogenic activities (e.g. climate change, pollution) as well as natural sources (Noyes et al., 2009). This change will affect many factors that are crucial for amphibians, such as ambient temperatures, hydroperiod of breeding waters, and the quantitative and qualitative composition of food (Corn, 2005). Food quantity and quality supposedly is influenced either directly or indirectly by, for example, a change in water temperatures and pH level, increasing UV radiation, increasing pollution and monotonization of aquatic and terrestrial habitats (Henrikson, 1990; Pierc and Montgomery, 1989). The ability to exhibit a high phenotypic plasticity provides an advantage in new and more unpredictable and variable habitats (Agrawal, 2001). Since temperature changes associated with climate change also alter the qualitative chemical composition of diets for larval amphibians, intestinal plasticity may play a key role in adapting to new diet compositions. Carreira et al. (2016) demonstrated that omnivorous amphibian tadpoles avoid protein-rich diets at higher temperatures simulating heat waves, and two out of three species benefited from this diet shift. *R. temporaria* tadpoles may thus optimize energetic intake by increasing herbivory (i.e. behavioral plasticity) and exhibiting intestinal plasticity at higher temperatures. However, strong declines in population sizes in this species have also recently been recorded (Neveu, 2009; Kwet, 2005). Furthermore, larval diet can affect bacterial communities in the guts of tadpoles, despite an effect on gut morphology. Knutie et al. (2017) demonstrated that a plant-based diet during the larval stage increases the susceptibility to pathogens in later life stages. A low-protein diet is also known to positively impact gut microbiota composition and microbial metabolites in vertebrates (Madsen et al., 2017; Ma et al., 2017; Zhao et al., 2019) and thus decrease the susceptibility to pathogens. Consequently, a diet shift to a low-protein plant-based diet at warmer temperatures could be acquired by digestive and trophic plasticity, but might also impact the infectious disease risk. Since little is known generally about phenotypic plasticity in the wild (Loman, 2002) and how flexibility during larval stage might influence fitness in later life stages, more long-term studies in natural environments are needed to understand how amphibians might cope with environmental changes.

## MATERIALS AND METHODS

### Experimental procedure

Eight egg clutches of *R. temporaria* were collected in April 2011 from a natural pond within the city limits of Hamburg, Germany (‘Volksdorfer Wald’, 53°6477 N, 10°1436 E) and transported to the laboratory at the University of Hamburg. Each clutch was kept separately in an aquarium until the embryos hatched and reached developmental stage 25 (i.e. no external gills visible, operculum developed, start of exotrophic feeding, Gosner, 1960). From each clutch, 20 tadpoles were randomly selected, and ten of these tadpoles were randomly assigned to either the high- or low-quality food treatment and subsequently raised in sibling groups in aquaria (16.8 cm×23.9 cm, volume 9.5 l), resulting in a total of 16 aquaria (eight clutches×two treatments) with ten tadpoles each. The experiment was conducted in a climatized laboratory with an ambient temperature of 13°C, a light regime of 11 h:13 h (day:night), using dechlorinated aged tap water (pH between 7.0 and 7.5) and with water changes in intervals of 4 days. When tadpoles reached developmental stage 36 (i.e. well-developed external hind legs with five distinguishable toes, Gosner, 1960), one tadpole was randomly selected from each aquarium, euthanized by immersion in MS222 solution and subsequently stored in 70% ethanol.

### Experimental treatments

Tadpoles were raised either under a low- or a high-quality food treatment. Low-quality treatment tadpoles were fed *Spirulina* algae (cyanobacteria) (JBL Premium flakes, JBL GmbH and Co. KG, 67141 Neuhofen, Germany). High-quality treatment tadpoles were fed *Daphnia pulex* (Tetra Delica Daphnien, Tetra GmbH, 49304 Melle, Germany). Food was provided *ad libitum*. Tadpoles were fed every day, with 0.3 g (early development) and 0.5 g (late development) food per aquarium, respectively, on the first day after water change, and then additional adding of food *ad libitum* in the following days of a water change interval. Prior to the experiments, both food types were powdered to provide an identical mechanical texture of food to restrict the differences between food types to their chemical composition. The differences in chemical composition of both food types refers to different protein, N and energy contents. We measured these variables and confirmed differences between *Spirulina* and *Daphnia* food: protein content, 35% versus 54%; N content, 5.6 versus 8.6%; energy content, 1256 kJ/100 g versus 1503 kJ/100 g, respectively. N content was quantitatively analyzed by the Kjeldahl method, and was converted accordingly to protein content N-factor 6.25. Energy content was analyzed by bomb calorimetry (6200 Isoperobol Calorimeter, Parr Instruments, Moline, Illinois). Analyses were done at the laboratory for chemical analyses at University of Hamburg.

### Morphometrical measures

Body length from each specimen was measured as length from snout tip to vent (McDiarmid and Altig, 1999) and developmental stage was determined (Gosner, 1960). As oral structures, maximum oral disc width, number of denticles present in 2nd posterior denticle row and total number of oral papillae (McDiarmid and Altig, 1999) were determined. As intestinal structures, gut length (from the end of the *Manicotto glandulare* to the vent), average intestinal diameter (calculated from five measurements uniformly distributed over the length of the intestine), and *Manicotto glandulare* volume [calculated as (0.5×diameter *Manicotto glandulare*)²×π×length *Manicotto glandulare*]. Fixation in alcohol had the effect that the intestines turned rigid and broke into parts when dissecting. We carefully sorted (from anterior to posterior) and measured those parts for length and the other variables, and then added the measurements. All measurements were taken on a digital microscope (Keyence VHX-500F) using integrated measuring software tools. During measurements, tadpoles or dissected intestinal structures were placed in a wax bowl with a dark background. For measuring variables of the oral apparatus, a pin was inserted through the oral disc and pushed through the head to fixate and open the oral disc. Moreover, oral papillae were stained with Methylene Blue.

### Intestinal microstructures

As intestinal microstructures, mean length of microvilli (average of five randomly selected microvilli) and density of microvilli (microvilli/µm^2^; average of five randomly selected 1 µm^2^ areas) were determined from two specimens of each experimental treatment. A 2–4 mm piece from the middle part of the gut was cut out, sliced open along its length with a razor blade, and spread out. This structure was then converted to 100% ethanol by a series of solutions with increasing ethanol concentrations, and subsequently dried by critical point drying. Dried samples were fixed to an object plate, existing rests of gut contents were removed with a fine brush, and the sample was vapor-coated with gold. Pictures of the intestinal surface were taken with a scanning electron microscope (LEO 1525 Gemini) at the Department of Electron Microscopy at the University of Hamburg, in a magnification between 2600 and 47,000 fold and a resolution of 2048×1536 pixels. Scanning electron microscopy was restricted to two individuals of each treatment. Therefore, these results were analyzed descriptively, and not statistically.

### Statistical analysis

All response variables (i.e. oral and intestinal structures) are morphometric variables that are usually highly dependent on body size. To account for the effect of body size, therefore, we used second-order statistics, i.e. we calculated residuals from linear regressions of the respective variable with body length using the full dataset (i.e. both treatment groups). Accordingly, for example, a specimen with a positive residual of gut length represents one with a relatively long intestine (in respect to its body size). We tested for hypothesis 1 (plasticity in intestinal structures) by comparing the residuals of gut length, average intestinal diameter and *Manicotto glandulare* volume between low- and high-quality food treatments using Mann–Whitney non-parametric tests. Here, sample size in the low-quality treatment was reduced to six as two specimens accidentally desiccated. Differences in intestinal microstructures (hypothesis 2) between the treatments were analyzed descriptively and visually due to the low number of replicates (*n*=2). We then tested hypothesis 3 (plasticity in the oral apparatus) by comparing the residuals of both number of denticles present in 2nd posterior denticle row and total number of oral papillae between low- and high-quality food treatment using Mann–Whitney non-parametric tests. We also compared for differences using absolute values of number of denticles present in 2nd posterior denticle row and total number of oral papillae, as these structures might be independent of body size (*n*=8 for both groups). All statistical tests were performed using SPSS 23.0 (IBM SPSS Software, Armonk, NY, IBM Corp.).
